# In vitro antitumor efficacy of hyaluronic acid coating for curcumin-loaded zeolitic imidazole frameworks-8 (ZIF-8) versus that of uncoated curcumin-loaded ZIF-8 nanocomposites

**DOI:** 10.1038/s41598-026-48707-9

**Published:** 2026-06-07

**Authors:** Walaa W. Oransa, Rasha F. Zahran, Rana R. El Sadda, Elshahat A. Toson

**Affiliations:** Department of Chemistry (Biochemistry Division), Faculty of Science, Damietta University, New Damietta, Damietta 34517 Egypt

**Keywords:** Hepatocellular carcinoma, Breast cancer, Hyaluronic acid (HA), Curcumin (C), Zeolitic imidazole frameworks (ZIF-8), Curcumin-loaded ZIF-8 (CZIF-8), Curcumin-loaded ZIF-8 coated with hyaluronic acid (CZIF-8/HA), Biotechnology, Cancer, Drug discovery, Oncology

## Abstract

Cancer continues to pose one of the biggest health burdens. Liver cancer has difficulties with early detection, efficient treatment and treatment resistance. Zeolitic imidazole framework-8(ZIF-8) is a type of metal organic frameworks (MOFs) that has a lot of useful properties; including its structural flexibility, high porosity and adjustable pore sizes, these make such framework good for drug delivery for hepatocellular carcinoma (HCC) and breast cancer. In the present study, curcumin was loaded into ZIF-8, and hyaluronic acid was applied as coat in a step-by-step, molar ratio, and pH-dependent manner. The resulting curcumin-loaded ZIF-8 (CZIF-8) and curcumin-loaded ZIF-8 coated with hyaluronic acid (CZIF-8/HA) nanocomposites were characterized by zeta potential, SEM, TEM, and PXRD. The obtained irregular semi-spherical nanocomposites have an average particle size of 127.93 and 131.07 nm; respectively. Also, their anticancer activities against two cancerous cell lines (HepG-2 and MDA-MB-231) and non-cancerous cell line (MCR-5) were assessed in vitro along with reference drug such as doxorubicin using the MTT assay. Additionally, the mechanisms by which these nanocomposites kill these tumor cells were found to involve apoptosis (elevated caspase-3 expression) rather than necrotic one and cell cycle arresting at G1/S phase. These particles have lower IC_50;_ particularly against HepG-2. Further, CZIF-8/HA demonstrated significantly higher cytotoxicity against cancer cells (HepG-2 and MDA-MB-231) compared to CZIF-8 and doxorubicin, while exhibiting minimal toxicity toward normal MRC-5 cells. These results imply that ZIF-8/HA is a promising nanocarrier for curcumin administration and could be used as a therapeutic strategy for breast and liver cancer treatment.

## Introduction

Cancer causes millions of deaths worldwide. It exhibits a wide range of characteristics and clinical symptoms. Most common cancer kinds were reported in men; including liver, colorectal but breast cancer in women is the most^1^. Breast cancer is the second most frequent invasive cancer in women and the second largest cause of cancer-related mortality. Because of metastasis and recurrence, the overall 5-year survival rate for patients with breast cancer remains below 10% due to its high incidence and fatality rate^2^. Hepatocellular carcinoma (HCC) generally affects men more than women. Further, bout 75% of all primary liver cancers are HCC^3^. Therefore, developing new HCC drugs which could be effective for these two types of cancers are urgently needed.

Curcuma longa’s rhizome contains curcumin, a naturally occurring photochemical that may be able to fight cancer^4^. The main reason for these traits is that it can make hydrogen bonds and go through rotamerization. Using curcumin, we need to make effective target-specific drug delivery systems (DDSs) that can specifically target cancer cells and deliver drugs in a way that lasts and breaks down the matrix. Curcumin is not suitable for direct therapeutic applications because it does not dissolve well in water, is not stable, breaks down quickly at physiological pH, and does not have a specific bio distribution^2,5^.

Metal organic frameworks (MOFs) are a type of porous crystalline structure made up of metal ions and organic ligands. They are getting a lot of attention lately as a possible new DDSs platform. This is because they are made up of many different things, can change shape and size, and have large surface area pores that can be changed to fit the need^6–8^.

Zeolitic imidazole framework-8 (ZIF-8) is one of the most promising subclasses of metal organic frameworks that have been studied a lot for use in drug delivery systems (DDSs), which is composed of biocompatible zinc ions and 2-methylimidazole. Its remarkable chemical and thermal stability, high porosity, large specific surface area, and ease of preparation at the nanoscale make it an excellent choice^9,10^.Because it breaks down progressively in acidic solutions but remains stable in neutral or alkaline solutions, its biodegradability at pH less than 6^11–13^, this give advantageous for targeted medication release in tumors^14–16^.Even with ZIF-8’s benefits, there are still some restrictions on their ability to be used in further clinical settings. These include their ease ability to polymerize and their inability to dissolve in water. All together causes them to be poorly biocompatible surface, aggregate in physiological settings and prevents their passive targeting. More reports have also raised concerns about ZIF-8’s possible toxicity. In order to get over these restrictions, some research has indicated that altering the surfaces of ZIF-8 to improve its biocompatibility, its hydrophilicity, its stability, and particularly, its functionality may be crucial to be used as DDS^12,17^.

The naturally occurring hydrophilic biopolymer; hyaluronic acid (HA), not only has negative charges but also a number of beneficial properties which include its non-immunogenicity, biocompatibility, biodegradability, and a special affinity for cell-specific surface clusters of differentiation 44 (CD44), which are overexpressed by many tumor cells during their growth^17,18^. According to Li et al.^19^, HA was added to the surface of ZIF-8 nanocomposites loaded with curcumin through a coordinated interaction may increase the solubility, stability and biocompatibility. All of these enable these nanocomposites to reach tumor sites and release the medication through the detachment of HA and the degradation of ZIF-8 under the influence of the acidic pH at the cancer tissues but not at the normal ones.

To elucidate the impact of the HA coating on the nanocarriers or nanocomposite (ZIF-8) and to combine the benefits of curcumin-loaded ZIF-8(CZIF-8) in the treatment of liver and breast cancer. Curcumin loaded ZIF-8/HA nanocomposites (CZIF-8/HA) was created by assembling HA on the particle surfaces via electrostatic interactions between carboxyl groups and zinc ions, following the synthesis of CZIF-8 based on the pH-dependent solubility of curcumin^20–22^.

The physical properties of the nanocomposites were assessed using zeta potential and electron microscopy (TEM and SEM), while structural features were examined by X-ray diffraction. Drug loading capacity (DLC %) and encapsulation efficiency (EE %) were utilized to assess the incorporation of curcumin within the ZIF-8 nanocarrier. In vitro cytotoxicity of CZIF-8 and CZIF-8/HA nanocomposites will be evaluated against the growth of human cancer cell lines; MDA-MB-231, and HepG-2 and also against non-carcenous lung fibroblast cells (MRC-5).The tested nanocomposites′ cytotoxicity was compared with the cytotoxicity of doxorubicin as a reference anti-tumor drug. Furthermore, the mechanism underlying this cytotoxicity was examined using the outcomes of caspase-3 measurement (ELISA), annexin staining technique as well as using flow Cytometry to study cell cycle arrest.

Although HA-coated ZIF-8 systems for curcumin delivery have been reported, this study presents several key advancements. Curcumin loading and HA coating were optimized via a stepwise, molar ratio- and pH-dependent approach, producing nanocomposites with controlled size, surface charge, and morphology. We further linked these properties to selective cytotoxicity against HepG-2 and MDA-MB-231 cells while sparing normal MRC-5 cells, and elucidated the mechanism of cell death via apoptosis and G1/S cell cycle arrest. Building on these insights, this study aims to compare the cytotoxicity of coated and uncoated CZIF-8 nanocomposites across two cancerous cell lines (HepG-2 and MDA-MB-231) and a non-cancerous cell line (MRC-5) in vitro, along with the toxicity of doxorubicin as a reference anti-tumor drug, with a focus on cytotoxic selectivity and apoptosis-related biomarkers. These combined methodological and mechanistic insights distinguish our work from previous studies.

## Materials and methods

### Reagents

2-Metheyl imidazole (99%) was acquired from Sisco Research Laboratory Pvt. Ltd.; sodium hyaluronate (500–750 kDa), Curcumin (> 98% pure), Dimethyl sulfoxide (DMSO), MTT, and trypan blue dyes were obtained from Sigma-Aldrich Co. (Saint Louis, USA); Zinc nitrate hexahydrate (Zn(NO3)2⋅6H2O, 99%) from Tianjin Kemiou Chemical Reagent, China; Fetal Bovine serum, DMEM, RPMI-1640, HEPES buffer solution, L-glutamine, gentamycin, and 0.25% Trypsin-EDTA from Lonza (Belgium) and 4’,6’-diamidino-2-phenylindole (DAPI) from Invitrogen, Life Technologies, Darmstadt, Germany. There was no need for any extra purification because all of the chemicals were of analytical grade.

### Synthesis of curcumin encapsulated in zeolitic imidazole frameworks (ZIF-8) nanocomposites (CZIF-8)

After thoroughly dissolving 8.22 g of 2-methylimidazole in 20 mL of distilled water, the solution pH was adjusted to approximately 11. The basic environment was achieved by 2-methylimidazole itself, which acts both as the organic linker and as a weak base in the reaction system. Subsequently, 0.005 g of curcumin was added to the mixture and stirred until completely dispersed^23^. Thereafter, 2 mL of an aqueous solution containing zinc nitrate hexahydrate (0.3 g) was added dropwise under vigorous stirring for 10 min. The solution immediately formed an orange precipitate, which was aged in the dark at 25 °C for 1 h. The obtained particles were collected by centrifugation at 10,000 rpm for 10 min and washed three times with absolute methanol. Finally, the precipitate was vacuum-dried at room temperature^17^.

### Synthesis of curcumin encapsulated in zeolitic imidazole frameworks (CZIF-8) nanocomposites coated with hyaluronic acid (CZIF-8/HA)

For 30 min, in the dark at 25 C^o^, 0.25 g of CZIF-8 nanocomposites was stirred gently in a solution of sodium hyaluronate (0.0005 g/ml). To get rid of extra hyaluronic acid, the solution was centrifuged for 15 min at 8000 rpm and then rinsed gently and briefly with pure methanol. Then; the particles were produced as before. Finally, they were all held at -20 °C^17^. CZIF-8/HA nanocomposite was stored at − 20 °C in airtight containers protected from light to preserve long-term stability. For short-term experimental use at room temperature, no observable changes in appearance or performance were noted.

### Characterization of CZIF-8 and CZIF-8/HA nanocomposites

Zeta potential was measured using a Malvern Zetasizer ver.7.01. After sample preparation, they were placed in cuvette and the scatter intensity was measured at 25 °C and measurements were taken in triplicates, then they were averaged.

The transmission electron microscope (TEM, Model Talos L120CG2-TEM-ThermoFisher-Europe) was used to measure the particle sizes of the two nanocomposites. The samples were diluted with distilled water using a sonicator for half an hour and then dropped onto a copper grid coated with carbon to create a thin liquid film. The films on the grid were left to dry at room temperature overnight before being examined by the TEM and captured on camera.

Surface morphology was investigated using scanning electron microscope (SEM) analysis using gold coating inspection to accelerate voltages of 20 kV (JEOLJSM- 6510 LV).

The prepared powder nanocomposites were placed on stubs made of aluminum utilizing carbon tape and a tiny layer of gold sputter-coated on top of it before taking pictures. The generated samples’ structural anomalies were examined using X-ray diffraction pattern (PXRD) using a Shimadzu XRD-6000 diffraction meter (Shimadzu Corporation, Tokyo, Japan) equipped with Cu Kα radiation (λ = 1.54 Å).

### Evaluation of drug loading capacity (DLC)

In 2 M HCl, 11 mg of the resultant nanocomposites were dissolved and subsequently diluted with 2 ml of pure ethanol. The curcumin content was subsequently determined using UV–vis spectroscopy at 425 nm, based on a standard curve correlating absorbance with varying curcumin concentrations at the same wavelength. The encapsulation efficiency (EE %) and drug loading capacity (DLC %) were calculated using the subsequent equations (Equations a and b):^24^.

a $${\text{Drug loading capacity }}\left( \% \right) = {\text{Weight of drug in nanocomposites}}/{\text{Weight of drug loaded nanocomposites}}\; \times \;{\mathrm{1}}00$$

b $${\text{Encapsulation efficiency }}\left( \% \right) = {\text{Weight of drug in nanocomposites}}/{\text{Total weight of initial feeding drug }} \times \;{\mathrm{1}}00$$

### Cell line propagation

This study utilized two cancerous cell lines, human hepatocellular carcinoma (HepG-2) and human breast cancer cells (MDA-MB-231), together with one non-cancerous normal human lung fibroblast cell lines (MRC-5). These cell lines came from the American Type Culture Collection (ATCC) in Rockville, MD. The RPMI-1640 medium had 50 µg/ml of gentamycin and 10% inactivated fetal calf serum added to it to help the cancerous cells grow. We grew MRC-5 cells in Dulbecco’s modified Eagle’s medium (DMEM) with 50 µg/ml of gentamicin, 10% heat-inactivated fetal bovine serum, 1% L-glutamine, and HEPES buffer. The temperature for all cell types was kept at 37 °C in a humidified environment with 5% CO_2_. They were subcultured every two or three weeks.

### In vitro evaluation of anticancer activity of CZIF-8 and CZIF-8/HA nanocomposites using MTT assay and their therapeutic indices

We used the MTT assay to test the anti-proliferative activity against two cancerous cell lines (HepG-2) and (MDA-MB-231) and the non-cancerous normal human lung fibroblasts cell line (MRC-5). The test lasted for 24 h. Along with doxorubicin as a positive control, apoptosis, cell cycle analysis, and Caspase-3 were used to look at how CZIF-8 and CZIF-8/HA nanocomposites worked against cancerous cell lines.

In the antitumor activity assay, HepG-2 and MDA-MB-231 tumor cell lines were seeded into Corning 96-well tissue culture plates at a density of 5 × 10⁴ cells per well and incubated for 24 h. Subsequently, the tested nanocomposites were added in triplicate across twelve serial concentrations for each compound. For each plate, six wells containing either culture medium or 0.5% DMSO served as vehicle controls. The normal cells were also put into a 96-well plate with 100 µl of growth medium and 1 × 10^4^ cells per well. After seeding for 24 h, fresh medium with different amounts of the test nanocomposites was added. Using pipette with many channels, we added serial two-fold serial dilutions of the tested nanocomposites to confluent cell monolayers that were inserted into 96 well, flat-bottomed microtiter plates (Falcon, NJ, USA). These plates were kept at 37 °C in a humidified incubator with 5% CO_2_ for 24 h. Three wells were utilized for each concentration of the tested nanocomposites. Control cells were incubated without any nanocomposites or DMSO. The small amount of DMSO in the wells (at most 0.1%) did not affect the results of the experiment. Subsequent to culturing the viable cells, a colorimetric technique was employed to determine the yield^25^.

MTT assay was used to detect the number of viable cells following incubation for 24 h. The media in the 96 well plates was taken out and replaced with 100 µl of new RPMI 1640 culture medium that did not contain phenol red. Then, 10 µl of the 12 mM MTT stock solution in PBS was added to each well, even the ones that weren’t treated. The 96 well plates were kept with 5% CO_2_ for 4 h at 37 °C. Then, 85 µl of media from the wells was taken out, and 50 µl of DMSO was put into each one instead. The solution was mixed well and kept for 10 min at 37 °C. A microplate reader (SunRise, TECAN, Inc, USA) was used to measure the optical density at 590 nm. The objective was to determine the number of viable cells and the corresponding percentage, calculated using the formula [(ODt/ODc)] x100%, where ODt represents the mean optical density of treated wells and ODc denotes the mean optical density of untreated cells^25^.

In order to obtain the survival curve for each cancerous cell line following treatment with nanocomposites, we show how the number of viable cells changes with the concentrations of the tested nanocomposites. This is by utilizing Graphpad Prism program (San Diego, CA, USA) to find the Cytotoxic Concentration (CC_50_) or Inhibitory Concentration (IC_50_), which is the amount of a substance that will kill half of the living cells^26^. The selectivity of the nanocomposites for neoplastic cells and its safety for healthy cells is referred to as the therapeutic index (TI). The value can be derived from the ratio of the CC_50_ of normal cells (MRC-5) to the IC_50_ of cancer cells (SI = CC_50_ MRC-5 / IC_50_ cancer cells)^27^.

### Evaluation the mechanism of cytotoxicity against (HepG-2) and (MDA- MB-231) treated cells

#### Cell cycle analysis using flow cytometry of (HepG- 2) and (MDA-MB-231) cell lines treated with CZIF-8 and CZIF-8/HA nanocomposites

CycleTESTTM PLUS DNA Reagent Kit (Becton Dickinson Immunocytometry Systems, San Jose, CA) was used to evaluate cell cycle analysis on the HepG-2 and MDA-MB-231 malignant cell lines to see how the nanocomposites affected them. The cells whether treated with the tested nanocomposites or not, were stained with propodium iodide stain (PIS) as directed by the kit and then examined with a cytometer. The Cell Quest program (Becton Dickinso Immunocytometry Systems, San Jose, CA) looked at how cells were spread out in the cell cycle^28,29^.

#### Apoptotic analyses (annexin V-FITC assay of HepG-2and MDA-MB-231 treated with CZIF-8 and CZIF-8/HA nanocomposites)

Apoptotic cells were later analyzed with the Annexin V-FITC assay. In summary, HepG-2 or MDA-MB 231 cells were cultured to a confluent monolayer (70–80% confluence) and subsequently exposed to the investigated nanocomposites at the IC50 concentration. Following 24 h of treatment, the cells were harvested and washed twice in Phosphate Buffered Saline (PBS) for 20 min each, then applying a binding buffer. Furthermore, both treated and untreated cells were resuspended in 100 µL of binding solution from the kit, augmented with 1 µL of FITC-Annexin V (Becton Dickinson BD PharmingenTM, Heidelberg, Germany), and incubated for 40 min at 4 °C. Thereafter, the cells were rinsed and resuspended in 150 µL of binding buffer, thereafter supplemented with µL of 4’, 6’-diamidino-2-phenylindole (DAPI) at a concentration of 1 µg/mL in PBS. The cells were later analyzed using the BD FACS Calibur flow cytometer (BD Biosciences, San Jose, CA)^28,29^.

### In vitro fold change of caspase-3 apoptotic protein expression level in (HepG-2) and (MDA-MB-231) cell lines treated with CZIF-8 and CZIF-8/HA nanocomposites using ELISA technique

To elucidate the potential apoptotic mechanism of action on tumorized cell lines, the levels of the apoptotic protein marker caspase-3 were evaluated using an ELISA colorimetric kit (Bio Vision Research Products, 980 Linda Vista Avenue, Mountain View, CA 94043, USA) in accordance with the manufacturer’s instructions. The cells were inoculated at a density of 1 × 10^5 cells/ml onto 6 well plates one day before to the experiment. Upon the establishment of a full monolayer cell sheet in each well of the plate, the tested nanocomposites were administered into a 6-well tissue culture plate at IC_50_ concentration. Each nanocomposite was executed in triplicate. Following incubation, cells were harvested using 0.25% trypsin; a 2 ml culture medium was included, and the resultant mixture was centrifuged at 1500 rpm for approximately 10 min at 4˚C in a Sigma refrigerated centrifuge, subsequently rinsed three times with PBS (pH 7.4). The pellet was placed into an extraction solution comprising 20 mM potassium phosphate buffer (pH = 7) and a protease inhibitor cocktail. The cells were subjected to sonication in ice cold normal saline (1/9, w/v) using a Virsonic ultrasonic cell disruptor for 10 min, followed by centrifugation at 5000 rpm for 5 min at 4˚C. The supernatant was subsequently stored at (-80˚C) until the experiments were conducted.

## Results

### Characterization of CZIF-8 and CZIF-8/HA nanocomposites

#### Zeta potential

**Fig. 1:**
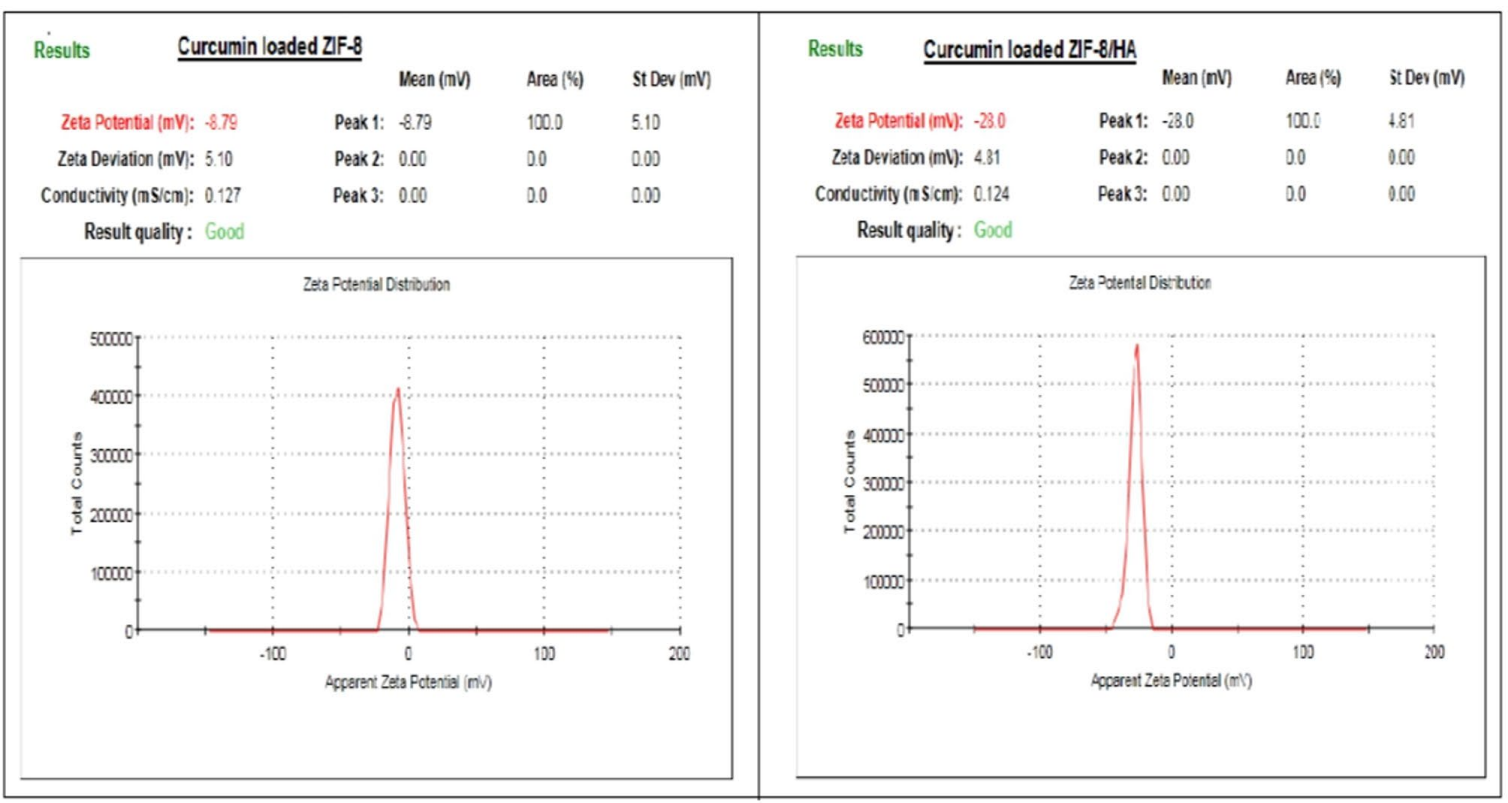
Zeta potential of CZIF-8 and CZIF-8/HA nanocomposites in water.

As shown in Fig. 1, the surface charge of the nanocomposites was assessed via zeta potential measurements, which are a key indicator of particle stability. Therefore, higher absolute values (either positive or negative) improved particle stability through stronger electrostatic repulsion, preventing aggregation. The zeta potential measurements for CZIF-8 and CZIF-8/HA were (-8.79 mV) and (-28.0mV) respectively.

### Transmission electron microscope (TEM)

**Fig. 2:**
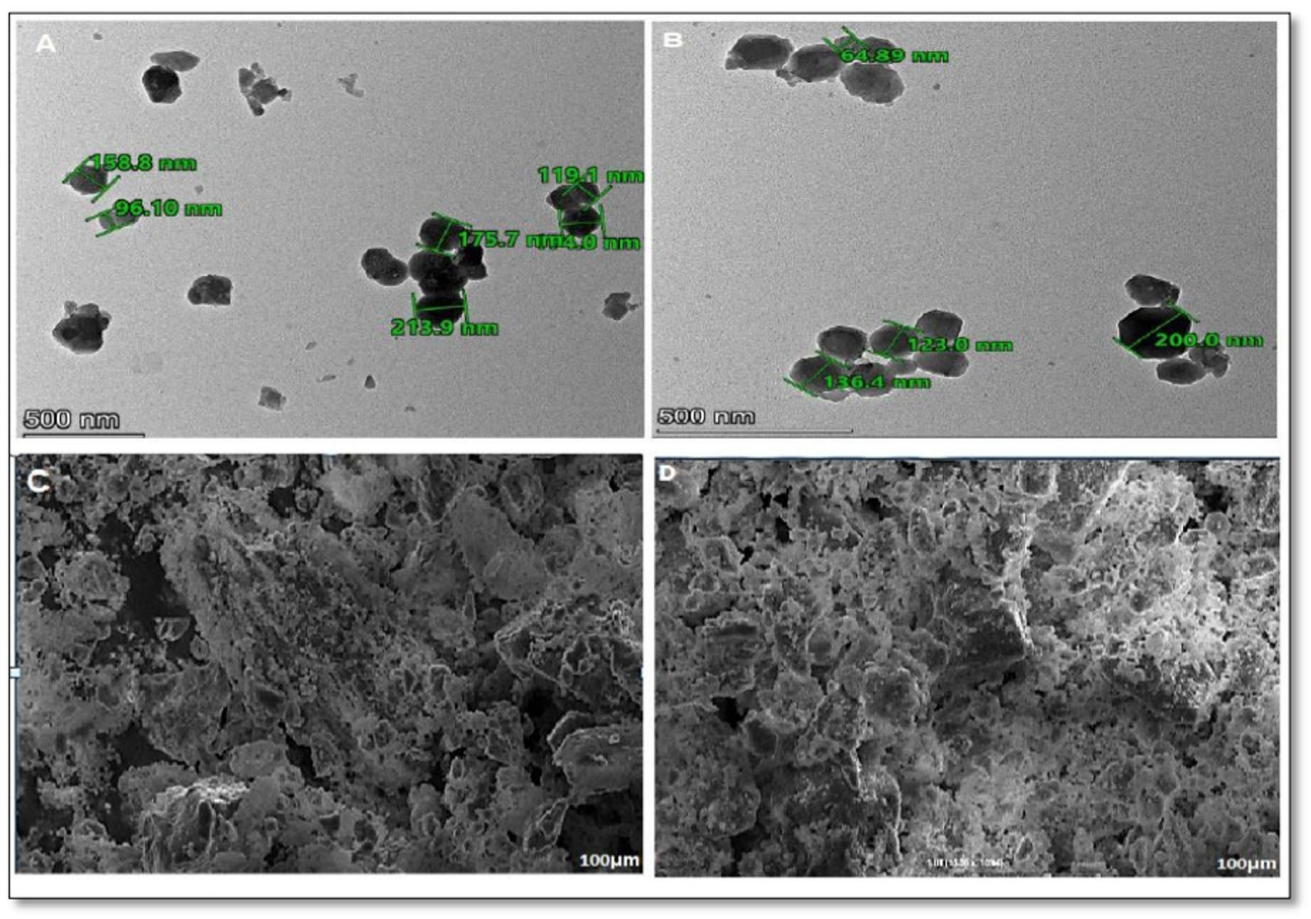
TEM images and SEM images of CZIF-8 and (**A**,**C**) and (**B**,**D**) for CZIF-8/HA nanocomposites respectively.

The shape and size of the nanocomposites were examined, as illustrated in Fig. 2A and Fig. 2B CZIF-8 with smooth surfaces and nearly irregular to semi-spherical shapes with a slightly agglomerated structure, showed a relatively broad size distribution. The majority of the particles appeared to be loosely aggregated or individual, with a mean size of (127.93 nm) and a range of (96.10 nm to 213.9 nm). The structure of ZIF-8/HA loaded with curcumin, on the other hand, is more aggregated and contains slightly bigger rough particles, with a mean particle size of (131.07 nm) and a range of 64.89 nm to 200.0 nm.

### Scanning electron microscope (SEM)

To assess how the addition of HA affected the structural integrity and surface characteristics of the produced nanocomposites, SEM imaging was used to look at their morphological characteristics. As shown in Fig. 2C and Fig. 2D CZIF-8 exhibits irregularly aggregated particles with rough, porous surfaces; the edges of the particles seem less distinct. Porosity and surface roughness are readily apparent in spite of the aggregation. The effective encapsulation of curcumin into ZIF-8 is demonstrated by the massive particle clusters and their interconnected structure. CZIF-8/HA, on the other hand, exhibits a more intricate and compact surface shape, suggesting a composite composition. There is less obvious porosity and the particles appear more fused and firmly packed.

### Powder X-ray diffraction (PXRD)

**Fig. 3:**
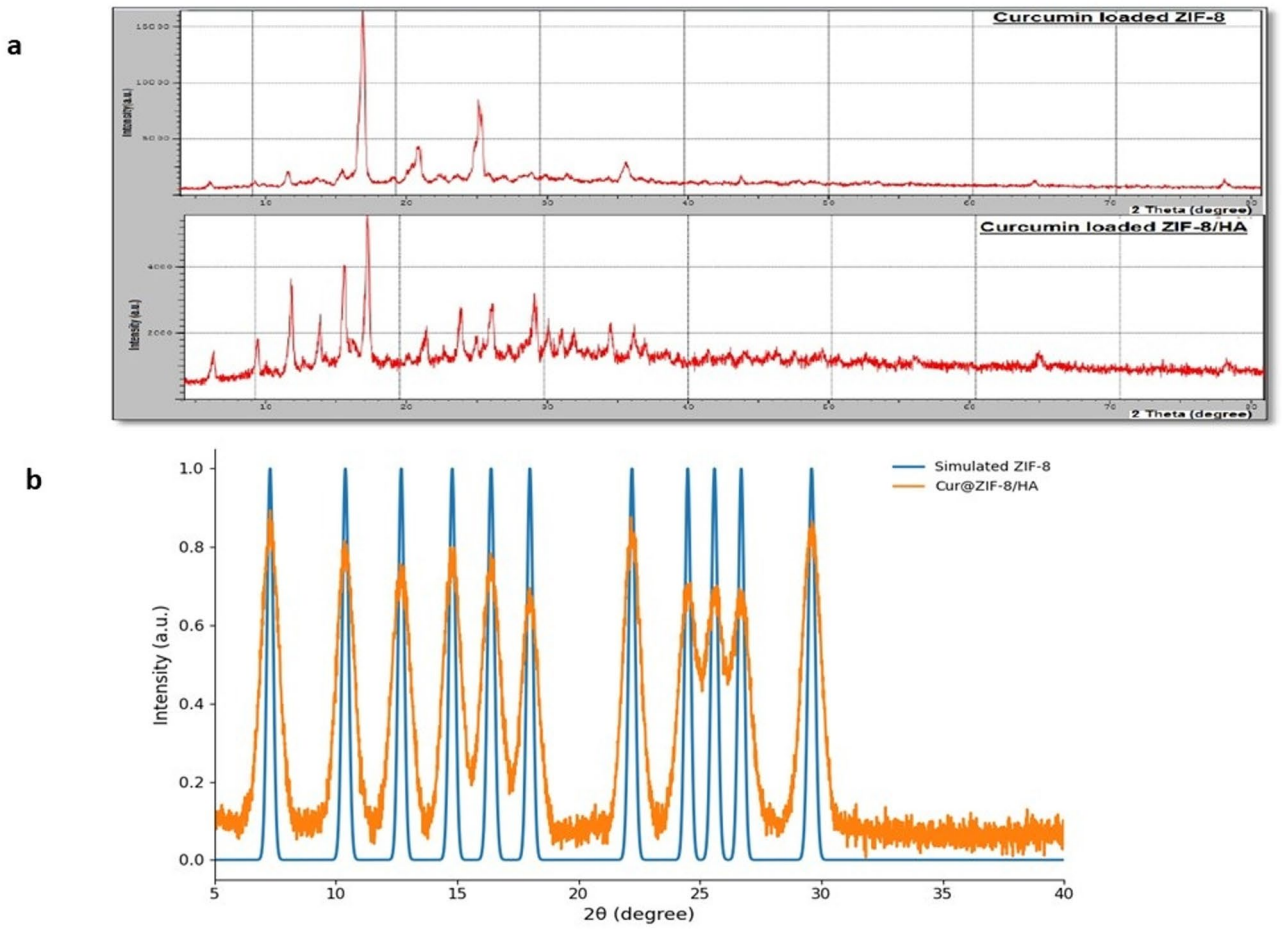
(**a**) PXRD spectra of CZIF-8 and CZIF-8/HA nanocomposites. (**b**) Overlay of a reference ZIF-8 PXRD pattern constructed from literature-reported diffraction peaks and the experimental CZIF-8/HA sample in the 2θ range of 5–40°. The characteristic reflections of ZIF-8 are retained, while peak broadening, reduced intensity, and baseline elevation after modification indicate partial surface amorphization and reduced crystallinity.

In order to confirm the crystalline nature of the prepared nanocomposites the PXRD patterns for both nanocomposites have peaks differ in sharpness, ranged from 2θ = 7 ^ο^ to 20 ^ο^) in Fig. 3a shows that CZIF-8 sample exhibits sharp and intense peaks at 2θ values around 17.6^ο^, 21.5^ο^ and 25.8^ο^ correspond to planes (1462), (658) and (327) respectively. The CZIF-8/HA sample, in contrast, has comparable peak positions at 17.6^ο^ and additional strong peaks at 12.4^ο^ and 16.1^ο^ correspond to planes (438), (259) and (278) respectively, but with much broader and considerably less intense peaks.

These observations indicate that the overall crystalline framework of ZIF-8 is preserved after curcumin loading and HA modification Fig. 3b, as no characteristic peaks disappear and their positions remain consistent. The peak broadening and intensity reduction can be attributed to partial surface amorphization, reduced crystallite size, and slight loss of long-range order upon functionalization. Such effects are consistent with previously reported studies on ZIF-8 and related MOFs following guest encapsulation and surface modification^24^. Overall, the PXRD results confirm successful incorporation of curcumin and HA while maintaining the integrity of the ZIF-8 sodalite structure.

### Drug loading capacity (DLC %) and encapsulation efficiency (EE %) of CZIF-8 and CZIF-8/HA nanocomposites

The drug loading efficacy of the resultant nanocomposites was assessed based on drug loading capacity (DLC %) and encapsulation efficiency (EE %). CZIF-8 demonstrated a drug loading capacity of 4.36% and an encapsulation efficiency of 57.25%. In contrast, CZIF-8/HA exhibited diminished values, with a DLC of 3.32% and a DLE of 44.37%.

### In vitro evaluation of anticancer activity of CZIF-8 and CZIF-8/HA using MTT assay

**Fig. 4:**
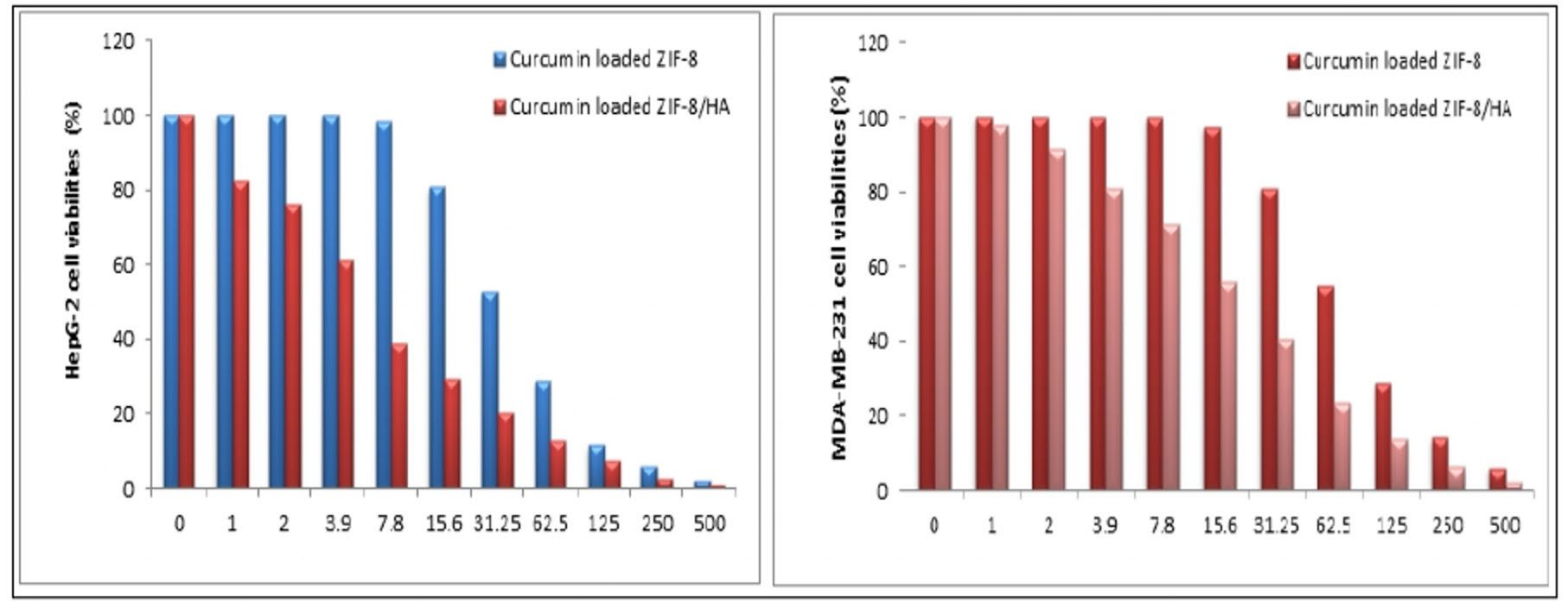
Relative cell viabilities of (**a**) HepG-2 and (**b**) MDA-MB- 231 cells incubated for 24 h with various concentrations of CZIF-8 and CZIF-8/HA nanocomposites.

The cell viabilities analysis of HepG-2and MDA-MB-231cells using MTT assay following 24 h incubation with various concentrations of CZIF-8 and CZIF-8/HA ranging from (0–500 µg/ml) as shown in Fig. 4, both nanocomposites demonstrated a dose-dependent cytotoxic effect on cells viability especially at lower concentrations (from 3.9 to 62.5 µg/ml). For HepG-2 cells the population viability decrease to 98.15% when treated with (7.8 µg/ml) CZIF-8 while CZIF-8/HA at the same dose effectively decrease cells viability to 38.56%.The maximum concentration (500 µg/ml) decrease cells viabilities to 1.98% and 0.79% for CZIF-8 and CZIF-8/HA respectively. Similarly, for MDA-MB-231cells the viability approximately dropped to 93.45% and 52.61% at (7.8 µg/ml) CZIF-8 and CZIF-8/HA respectively. At higher concentrations (125–500 µg/ml) both exhibit strong cytotoxic effect as CZIF-8 decrease cells viability to 3.29% and CZIF-8/HA to 1.35% at the maximum concentration (500 µg/ml).

**Fig. 5:**
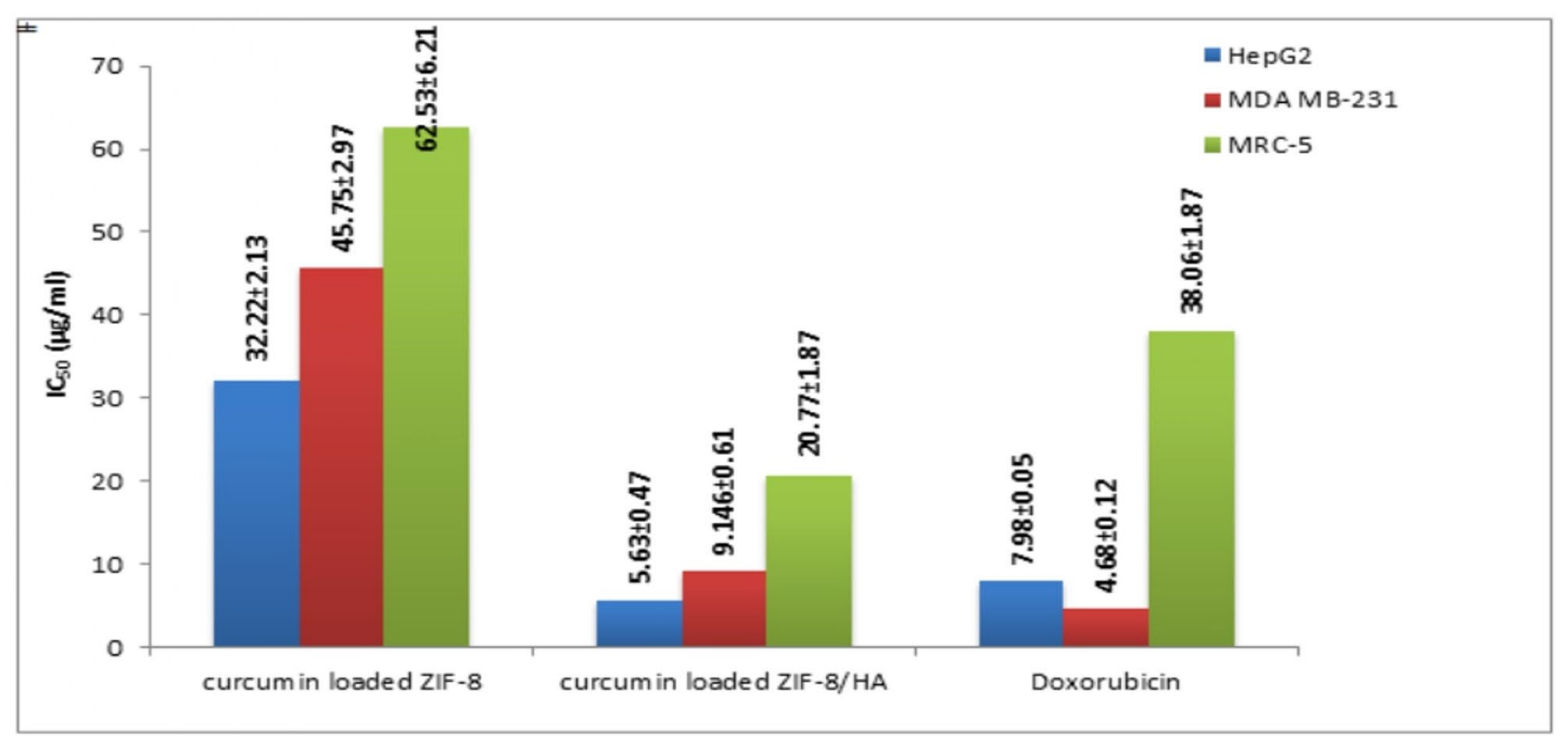
IC_50_ (µg/ml) for CZIF-8 and CZIF-8/HA nanocomposites and doxorubicin against two cancerous cells HepG-2 and MDA –MB-231 and normal cell MRC-5 at 24 h incubation.

The IC₅₀ values which were presented in Fig. 5 demonstrated the cytotoxic efficacy of nanocomposites, and doxorubicin as reference drug against two cancer cell lines HepG-2 and MDA-MB-231 and a normal human lung fibroblast cell line (MRC-5) following 24 h of incubation. In HepG-2 cells, CZIF-8/HA showed significantly enhanced cytotoxicity (IC₅₀ = 5.63 ± 0.47 µg/ml) compared to CZIF-8 (32.22 ± 2.13 µg/ml) while doxorubicin exhibits an IC₅₀ of (7.98 ± 0.05 µg/ml).Similarly, in MDA-MB-231 breast cancer cells, IC₅₀ values for CZIF-8/HA, CZIF-8 and doxorubicin were (45.75 ± 2.97 µg/ml), (9.15 ± 0.61 µg/ml) and (4.68 ± 0.12 µg/ml) respectively. In contrast, in the normal MRC-5 cells, both curcumin nanocomposites exhibited much higher IC₅₀ values (62.53 ± 6.21 µg/ml) for ZIF-8 loaded curcumin and (20.77 ± 1.87 µg/ml) for CZIF-8/HA compared to the cancer cell lines. Doxorubicin, however, showed a relatively lower IC₅₀ (38.06 ± 1.87 µg/ml) in normal cells.

### Therapeutic indices of CZIF-8 and CZIF-8/HA nanocomposites

**Fig. 6:**
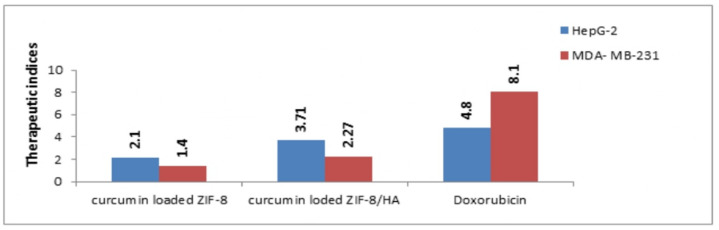
Therapeutic index (TI) values for three treatments CZIF-8, CZIF-8/HA, and doxorubicin on two cancer cell lines: HepG-2 and MDA-MB-231 after 24 h of incubation.

Therapeutic (TI) refers to the compound’s cytotoxic selectivity for cancer cells and its safety for healthy cells^27^. It can be calculated by dividing the IC50 value for cancer cells by the CC_50_ of normal cells (MRC-5) (TI = CC_50_ MRC-5/IC_50_ cancer cells) Fig. 5 .The value of the therapeutic index lower than 3 is recommended to have a high selectivity of the compound towards a particular cell line as High TI values imply malignant cells will be killed at a higher rate than normal cells^30^.The calculated TI varies from 1.4 to 8.1 Fig. 6**.**These value shows that CZIF-8 demonstrated TI values of 2.1 and 1.4 for HepG-2 and MDA-MB-231, respectively. In contrast, CZIF-8/HA exhibited TI values of 3.71 for HepG-2 and 2.27 for MDA-MB-231. Doxorubicin displayed TI values 4.8 and 8.1 for HepG-2 and MDA-MB-231, respectively.

### Cell cycle arrest promoted by CZIF-8 and CZIF-8/HA nanocomposites

**Fig. 7:**
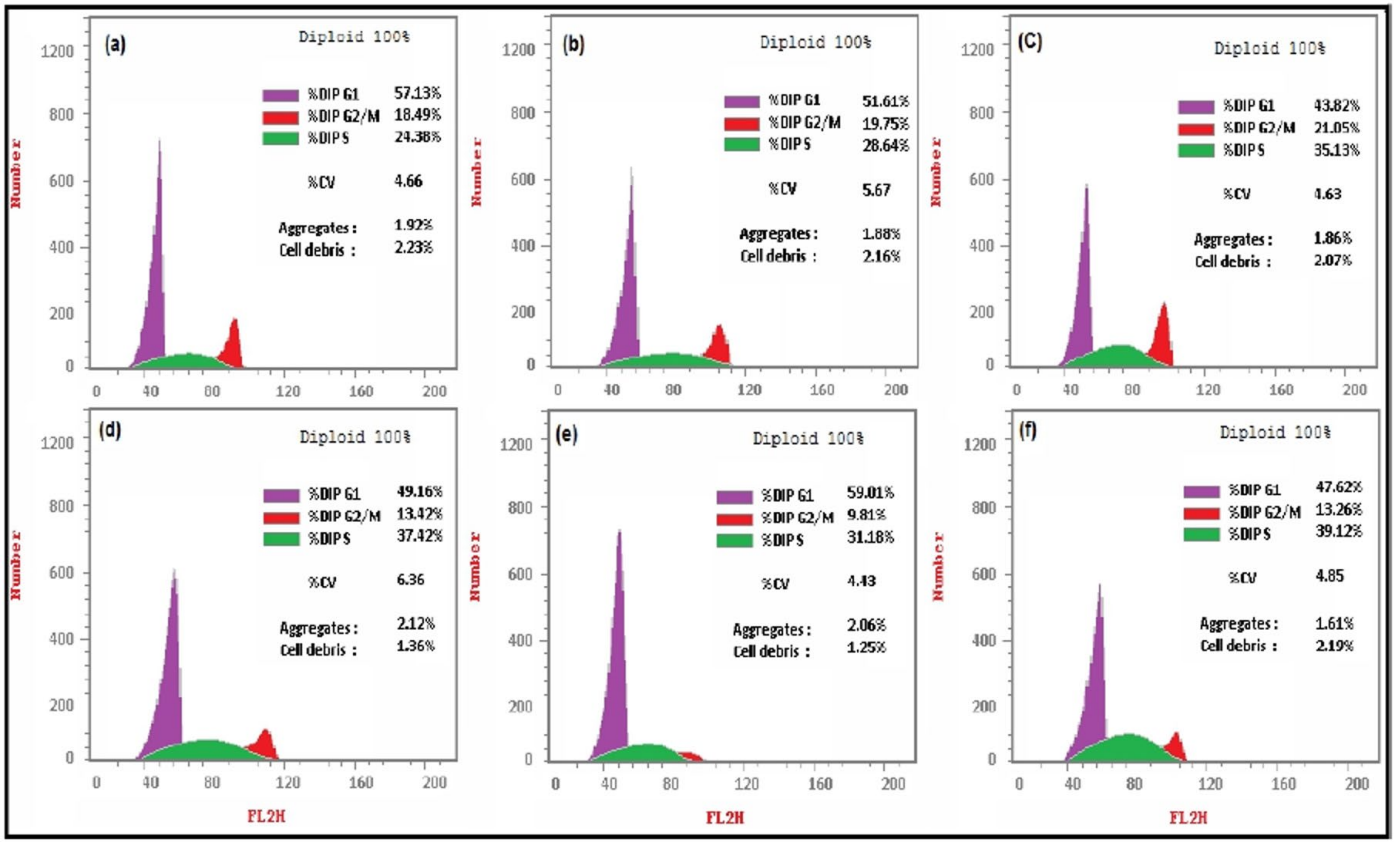
Cell cycle distribution in HepG-2 (**a**–**c**) and MDA-MB-23 cells (**d**–**f**): (**a**,**d**) cells treated with CZIF-8, (**b**,**e**) cells treated with CZIF-8/HA, and (**c**,**f**) untreated control cells.

Flow cytometric analysis which performed at IC_50_for 24 h to determine the impact of CZIF-8 and CZIF-8/HA on the cell cycle progression of both HepG-2 and MDA-MB-231carcenous cells. Figure 7 shows the plotting histogram of the cells population that distributed in the different cell cycle phases. The cell cycle comprises four primary phases: G0 (a quiescent, non-dividing state), interphase which includes the G1 (first growth state), S (synthesis), and G2 (second growth state) phases and the mitotic (M) phase. Cell division progresses sequentially from G1 to S, then G2, and finally M phase, resulting in DNA replication and the generation of two daughter cells.

For HepG-2 the untreated cells have a high percentage of cells which was observed in the S phase (35.13%), after treatment with CZIF-8, a notable increase in the G1 phase cells was observed (57.13%) with reduction in S phase cells (24.38%) and (18.49%) were found in G2/M. However, treatment with CZIF-8/HA also increased the G1 population (51.61%) but to a lesser extent than CZIF-8. On the hand, CZIF-8/HA lightly led to a lower proportion of cells in the S phase (28.64%).

Similarly, MDA-MB-231 untreated cells demonstrated a high percentage in S phase (39.12%). Treatment with CZIF-8 resulted in a reduction of G1 phase cells to (49.16%) and decrease in S phase cells to (37.42%). Likewise, CZIF-8/HA caused more evident G1 arrest (59.01%) and a slight decrease in S phase cells (31.18%) compared with untreated cells.

### Apoptotic effects of CZIF-8 and CZIF-8/HA

**Fig. 8:**
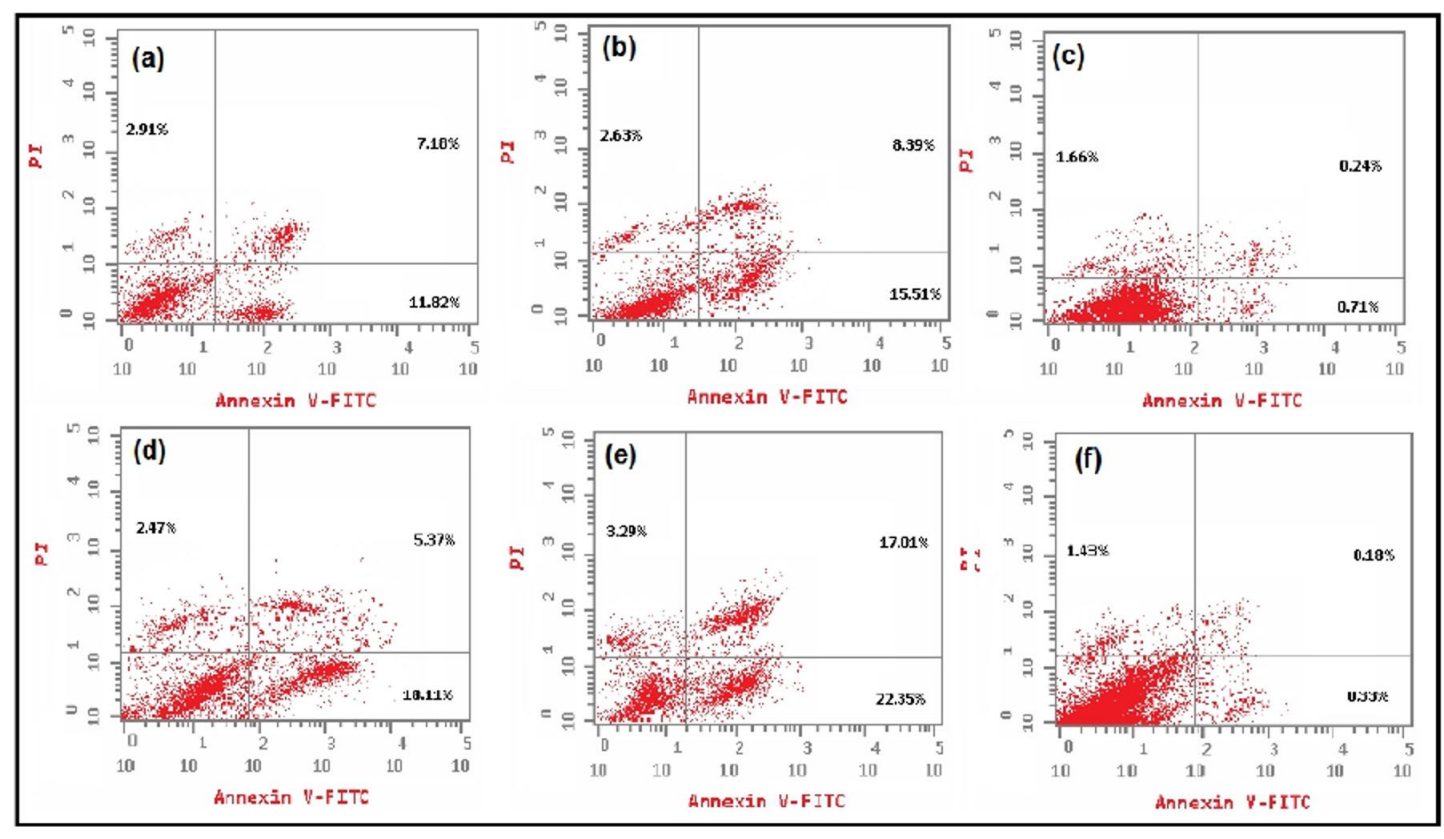
Apoptotic versus necrotic percentages in HepG-2 (**a**–**c)** and MDA-MB-23 cells (**d**–**f**): (**a**,**d**) cells treated with CZIF-8, (**b**,**e**) cells treated with CZIF-8/HA, and (**c**,**f**) untreated control cells.

As showed in Fig. 8, the HepG-2 and MDA-MB 231 cells were treated for 24 h with CZIF-8 and CZIF-8/HA under their IC_50_ values. After that, they were tested for apoptosis, necrosis and cell cycle analysis to demonstrate the cytotoxic effect of the prepared nanocomposites on the tested malignant cells using Annexin V-FITC assay. For HepG-2, treatment with CZIF-8 led to a significant increase in the total apoptotic cells percentage (19.0%). Early apoptosis was (11.82%) and those in late apoptosis was (7.1%.) late apoptosis. 2.91% necrotic cells were reported. On the other hand, CZIF-8/HA further enhances the apoptotic population (15.51% for early, and 8.39% for late apoptosis), resulting in a total of 23.9% for apoptotic cells death. The cells undergo necrotic represent only (2.63%) of the cells. The control group (untreated cells) showed negligible apoptosis as apoptotic percentage was 0.95% and (1.66%) for necrosis.

Similarly, in the MDA-MB-231 cell line, curcumin loaded CZIF-8 induced a total of 23.48% cellular apoptosis (18.11% early, and 5.37% late apoptosis) and (2.47%) for necrosis. Upon treatment with CZIF-8/HA, apoptosis was markedly increased to reach a (39.36%) of total apoptosis (22.35% early, and 17.01% late apoptosis) and (3.29%) for necrotic percentage. The untreated control cells; again, showed minimal apoptosis (0.51%) and 1.43%) for necrotic cells.

### Effect of CZIF-8 and CZIF-8/HA on the expression of the apoptosis-regulated caspase-3 protein

**Fig. 9:**
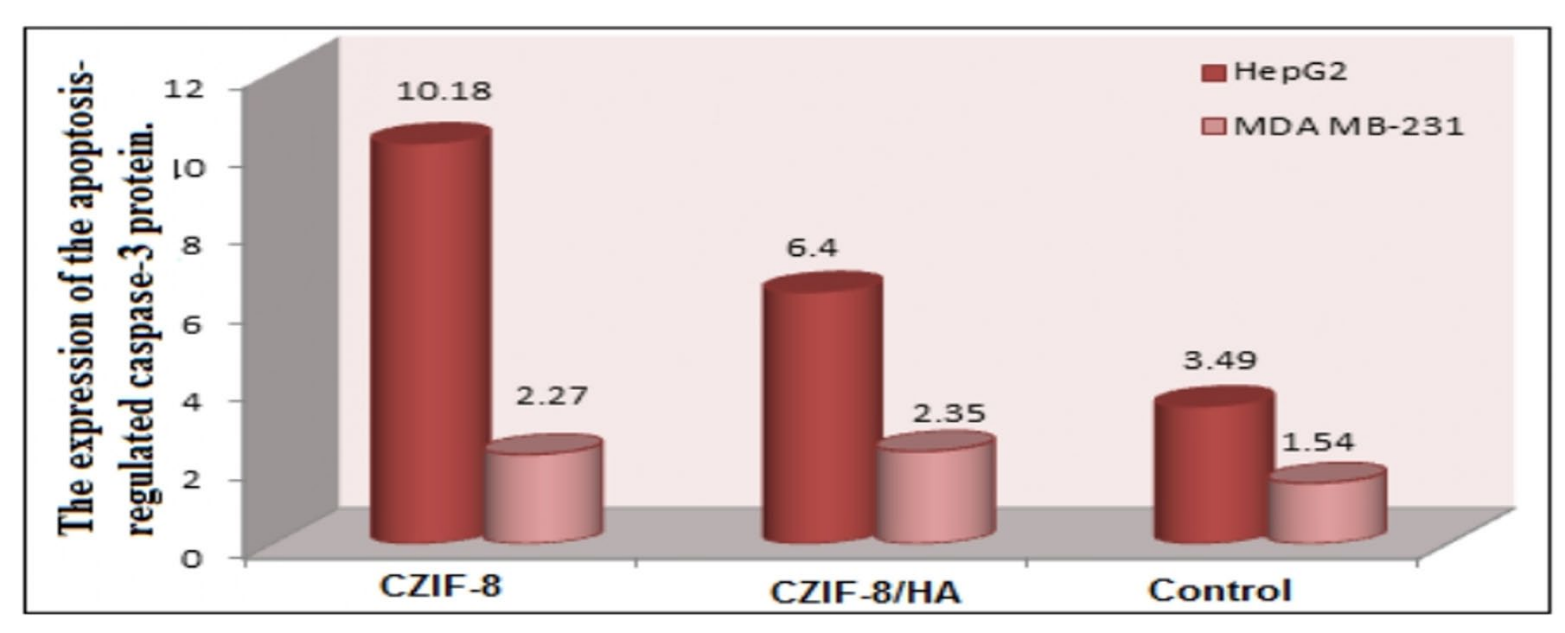
In vitro fold changes in apoptotic caspase-3 levels in HepG-2 and in MDA-MB-231 cancer cell lines.

In order to investigate the mechanism of apoptosis which may induced by nanocomposites on the tested cancer cell lines, we use ELISA technique to track the protein expressed by the apoptosis-regulated gene; namely, caspase − 3. The results were depicted in Fig. 9 significant upregulation of caspase-3 protein in both tumor cell lines after treatment with curcumin-loaded nanocomposites, indicating the induction of apoptosis. For HepG-2 cells, caspase-3 protein baseline level of the untreated cells was (3.49ng/ml). This was significantly increased to (6.4ng/ml) after treatment with CZIF-8/HA and reached a maximum of (10.18ng/ml) after CZIF-8 treatment. Similarly, in MDA-MB-231 cells, caspase-3 protein was elevated after treatment with CZIF-8 to reach (2.27ng/ml) and CZIF-8/HA reached (2.35ng/ml) when compared to that of the control cells (1.54ng/ml).

## Discussion

The manufacture of CZIF-8 nanocomposites was based on the basic principle that curcumin is soluble at different pH values^31^. The following steps were taken in order to improve the encapsulating of curcumin in nanoparticles forms. Firstly, the pH was elevated to an alkaline level, which dissolved the curcumin; next, the pH was dropped to an acidic level, which precipitated the curcumin. Consequently, curcumin was directly dissolved in 2-methelyimidazole aqueous solution as was carried out in this study at pH around 11^32^. Zinc ions were then added to ZIF-8 to create CZIF-8 and then the resulted orange nanocomposites were coated with hyaluronic acid (HA) in order to increase hydrophilicity of curcumin and its dispersity in aqueous medium. A molar ratio of 1:100 (zinc to 2-methylimidazole) was employed in the production of CZIF-8. The substantial excess of ligand is crucial for shifting the equilibrium towards the development of the zeolitic framework while inhibiting the emergence of impurity phases like Zn (OH)_2_^33^.The basic environment was established utilizing 2-methylimidazole itself which function as both the organic linker and the base. While OH ions are present and the rapid coordination between Zn²⁺ ions and imidazole nitrogen is significantly stronger than that of Zn(OH)_2_ under these specific molar ratio between imidazole and zinc ions (1:100).Thus, ZIF-8 framework was formed over Zn(OH)₂ precipitation, as widely reported for ZIF-8 synthesis^34^.

The capacity of the encapsulated curcumin was determined to be 3.32% for CZIF-8/HA, which is slightly lower than the value for CZIF-8 is 4.36%, similar to results obtained by Li et al.^19^ and Yu et al.^17^.The elevated DLC% and EE% values for CZIF-8 suggest minimal curcumin loss during the surface modification with HA, attributable to the inherent structural properties of ZIF-8, such as its high porosity, extensive specific surface area, and well-defined pore architecture, which facilitate effective drug incorporation^35^. Previous studies have demonstrated that curcumin is predominantly contained within the ZIF-8 framework during its production. The diminutive pore size of ZIF-8 (~ 3.4 Å)^36^, which is considerably less than the molecular dimensions of curcumin, prevents the drug from diffusing into existing pores, resulting in its physical entrapment during the in situ crystallization process. Alongside size constriction, secondary noncovalent interactions especially π–π stacking between the aromatic rings of curcumin and the imidazole linkers of ZIF-8 enhance the stabilization of the encapsulated drug within the framework^37^.

In contrast, the decreased DLC% and EE% values noted for CZIF-8/HA align with findings on surface-modified or coated MOFs systems. The deposition of a hyaluronic layer on ZIF-8 particles may obstruct pore entrances or diminish the available pore volume, consequently restricting curcumin diffusion into the internal framework after loading^19^. Furthermore, the supplementary mass from the HA coating elevates the overall weight of the nanocomposite without a corresponding rise in curcumin content, leading to a diminished estimated DLC%. Comparable decreases in drug loading metrics after the polymeric or inorganic surface coating of metal-organic frameworks MOFs have been extensively documented^12,38^.

The resulted nanocomposites were characterized by Zeta potential, SEM, TEM, and PXRD. Zeta potential results suggested that, increasing negative surface charge in the CZIF-8/HA system is attributed to the presence of HA, which introduces additional negatively charged functional groups on the nanocomposites surface compared to CZIF-8 alone. Colloidal literature firmly establishes that absolute zeta potential levels beyond approximately 25–30 mV (either positive or negative) signify robust electrostatic stability against aggregation^39^. This enhances its stability in biological fluids, making it a more reliable and effective carrier for the delivery of curcumin.

TEM results confirms the Irregular semi-spherical morphology and nanoscale particle size (100–200 nm) of CZIF-8 align with the findings of Zheng et al.^37^, as ZIF-8 generally forms polyhedral or near-spherical nanoparticles contingent upon synthesis conditions. Furthermore, the encapsulation of hydrophobic drugs, such as curcumin, within the ZIF-8 framework has been demonstrated to maintain the overall morphology while augmenting particle size due to curcumin interactions within the pores and on the surface. Increasing aggregation and the slight alternation in particle morphology and average size observed in CZIF-8/HA nanocomposites as the addition of HA seems to affect the overall particle structure, ionic interactions and intrinsic surface chemistry. The presence of HA may be the cause of clustering. Because of its hydrophilic and bioactive properties, HA may be potentially have improved antiparticle interactions, which could explain the observed particle agglomeration, This is consistent with the interpretations provided by Li et al.^19^.As a result, improvement in stability, biocompatibility, and possible targeting qualities are expected. These results show the morphological changes brought about by the addition of hyaluronic acid and validate the effective creation.

SEM analysis indicates that the rough and porous morphology of CZIF-8 aligns with previously documented ZIF-8-based drug delivery systems, wherein the encapsulation of curcumin results in partial surface coverage and particle aggregation^40^.In contrast, HA incorporation modifies surface architecture by producing denser, less porous particles. This morphological transformation suggests successful HA integration into the ZIF-8 matrix, likely due to HA’s tendency to form amorphous or layered structures that adhere to and fill the pores of ZIF-8 particles^41,42^.

The observed PXRD peaks for CZIF-8 align with the broader, attenuated peaks observed in the crystal structure of ZIF-8 documented by Tiwari et al.^24^.The retention of peak locations upon curcumin encapsulation does not modify the long-range order of the ZIF-8 peaks (2θ = 7–20°), corroborating prior studies that indicate the structural stability of ZIF-8 upon the inclusion of small-molecule drugs. Post-HA modification, the PXRD pattern preserves all distinctive ZIF-8 reflections without any peak displacement; nonetheless, the noted decrease in peak intensity and broadening aligns with surface coating effects and partial amorphization caused by HA deposition, rather than structural failure. A comparable reduction in diffraction intensity without phase change has been documented for ZIF-8 composites covered with HA^43^.

Using MTT assay, curcumin nanocomposites were tested for their cytotoxic effect against HepG-2and MDA-MB-231 cancerous cells and MRC-5 normal cells. The cytotoxic effect of CZIF-8/HA is much greater than CZIF-8 in both cancerous cell lines; especially in the case of HepG-2.This results suggested that, the incorporation of HA into the ZIF-8 framework enhances anticancer effect of the HA-containing composite and this may be attributed to HA bioactivity, which could be facilitate cellular internalization and support the sustained release of curcumin. Our findings are nearly in agreement with those of Li et al.^19^, who demonstrated that the incorporation of HA enhanced the cytotoxicity of curcumin-loaded ZIF-8 nanocomposites across a wider concentration range (12.5–100 µg/ml). These inconsistencies highlight the possibility that even slight differences in nanocomposites preparation methods can result in significant difference in biological activity. In this study incorporation of hyaluronic acid was the case.

CZIF-8/HA nanocomposites showed significantly much lower IC₅₀ values (5.63 µg/mL and 9.15 µg/mL) respectively for HepG-2 and MDA-MB-231. It was more potent than doxorubicin in HepG-2 cells and comparable in MDA-MB-231 cells. Additionally, ZIF-8/HA loaded curcumin showed relatively lower toxicity to normal MRC-5 cells than the uncoated CZIF-8, indicating higher selectivity and a better therapeutic index. In our in vitro viability assay doxorubicin showed higher TI, but known in vivo toxicities. This is what we will examine in vivo on HepG-2 model.

Therapeutic index (TI) values show that CZIF-8 demonstrated relatively low TI values compared with HA coated nanocomposites, indicating limited selectivity especially for the breast cancer model. In contrast, functionalization with HA significantly enhanced the therapeutic index in both cell lines. This improvement can be ascribed to functionalization with HA, which is known to be selectively bind to CD-44 receptors overexpressed on the surface of various tumor cells^32,42^, this may be facilate receptor-mediated endocytosis and improving intracellular drug accumulation. Doxorubicin displayed the highest TI values among the tested treatments. These results highlight doxorubicin’s potent cytotoxicity and high cancer selectivity, particularly against MDA-MB-231 cells. So, further, in vivo study is essential to fully assess the biocompatibility and therapeutic index of these formulations.

Unchecked proliferation and continuous advancement are hallmarks of the cancer cell cycle. Any alteration in the control of the cell cycle phase causes cancer cells to proliferate quickly. Therefore, stopping the unchecked cell cycle at different points is essential for treating cancer^44^. In the current study, the cell cycle was studied in vitro on HepG-2 and MDA-MB-231 cells-treated with CZIF-8 and CZIF-8/HA and after it’s loading in an equivalent dose using the flow cytometric assay. The cell cycle assay results showed that, after treatment the HepG-2 cells with CZIF-8, a notable increase in G1 phase cells was observed with reduction in S phase cells and G2/M suggesting induction of G1 phase arrest. However, treatment with CZIF-8/HA also increased the G1 population but to a lesser extent than CZIF-8. On the other hand, a lower proportion of cells in the S phase indicate G1/S phase arrest and inhibition of DNA during synthetic phase. In case of MDA-MB-231 untreated cell, a high percentage of cells in S phase. After treatment with CZIF-8, a reduction of G1 phase cells was observed indicating G1/S-phase arrest. Likewise, CZIF-8/HA caused more evidence on G1 arrest and a slight decrease in S phase cells compared with those of the untreated cells. All together indicates that the HA coated nanocarriers was more effectively halted the tumor cell cycle before DNA synthesis. Our findings suggest that both nanocomposites interfered with the regular distribution of the cell cycle in HepG-2 and MDA-MB-231 cells because they may interact with intracellular nucleic acid and proteins, which could contribute to the observed effect; however, direct binding was not assessed in this study. Cell death results from both interaction with cellular proteins and the activation of certain apoptotic enzymes, such as caspase-3^45,46^. However the effects varied depending on the phase. These diversities could be linked to the two cancer cell lines’ distinct intracellular trafficking strategies and the levels of HA receptor expression.

In order to delineate the mode of cell death which can occur through two major mechanisms apoptosis (programmed cell death) or necrosis (uncontrolled cell death), annexin V-FITC assay was used to demonstrate the cytotoxic effect of the nanocomposites on the tested cells. Our finding confirms that, treatment of HepG-2 and MDA-MB-231 cells with CZIF-8 and CZIF-8/HA led to a significant increase in apoptotic cells than necrotic ones. In this regard, CZIF-8/HA formulation induced a more pronounced apoptotic response in MDA-MB-231 cells compared to HepG-2 cells. The negligible apoptosis in the untreated cells clarify the ability of both nanocomposites to induce cell death via apoptosis mainly through early apoptosis not through necrosis.

Caspase- 3 is a crucial effector and most prevalent in family of caspases, widely concede for their triggered proteolytic functions in enforcing apoptosis in cells reacting to particular internal or extrinsic triggers of this type of cell death^47^. Apoptosis resistance is one of the most popular causes of carcinogenesis. Therefore, the majority chemotherapeutic agents mediate induction of apoptosis in cancer cells via different mechanisms. Thus, the dominant idea in many cytotoxic chemotherapeutic treatments for cancer has been the activation of caspases to cause apoptosis^36,37^, . In this study we use the ELISA technique to track the expression of the apoptosis-regulated caspase-3 protein. Our findings showed a significant increase of caspase-3 protein in both cell lines following treatment with curcumin-loaded nanocomposites, indicating the induction of apoptosis. In HepG-2 cells, caspase-3 baseline protein level of the untreated cells increases significantly with CZIF-8/HA and reached a maximum of with CZIF-8. This substantial elevation indicates a strong pro-apoptotic response, particularly with the ZIF-8 formulation lacking HA. These results suggest a more rapid or potent release of curcumin. Similarly, in MDA-MB-231 cells, caspase-3 protein level was elevated after treatment with both nanocomposites compared to control cells. This evaluation is low if compared to HepG-2 cells. However, CZIF-8 enhances apoptotic signaling, particularly in HepG-2 cells, where the response is more pronounced. HepG-2 cells’ greater apoptotic response in contrast to MDA-MB-231 might be the result of variations in innate sensitivity to curcumin-mediated apoptosis, internalization of nanocomposites, or cellular uptake efficiency. Interestingly, CZIF-8/HA preserved similar efficacy in MDA-MB-231 but marginally decreased caspase-3 activation in HepG-2, possibly as a result of HA-mediated targeting or possibly by inducing apoptosis through activation of another apoptosis-regulation genes^48^. Overall, our earlier findings indicate that the curcumin-loaded nanoformulations’ potential act as therapeutic agents for hepatocellular and breast carcinomas possibly via apoptosis induction and cell cycle arrest. A key limitation of the present study is the lack of in vitro drug release data and control experiments comparing curcumin delivery with and without ZIF-8 and ZIF-8/HA, which will be addressed in future work to more accurately correlate curcumin release with the observed cytotoxic effects and clarify the nanocomposites’ role.

## Conclusion

In summary, curcumin was loaded into ZIF-8 and subsequently coated with hyaluronic acid (HA) in a molar ratio (1:100:20), pH-dependent pathway was used to create biopolymer-decorated ZIF-8 that would enhance curcumin’s solubility, stability, and anti-cancer action. The CZIF-8 and CZIF-8/HA nanocomposites were characterized by means of SEM, TEM, PXRD and zeta potential. Their average particle size is 127.93 and 131.07 nm, respectively, and their shape is uneven and semi-spherical, with some variations in the surface texture, pores, and edges. In vitro cytotoxicity, apoptotic induction, and selective targeting of cancer cells revealed that, curcumin-loaded ZIF-8/HA had a greater cytotoxic effect on HepG-2 and MDA-MB-231 malignant cell lines, particularly HepG-2, than uncoated CZIF-8 and doxorubicin; the reference drug. Unluckily, CZIF-8/HA had little effect on normal cells. Consequently, CZIF-8 nanocomposites promoted apoptosis in HepG-2 cancerous cells by upregulating the expression of caspase-3 protein higher than in CZIF-8/HA. Both nanocomposites interfered with the normal cell cycle progression in cancer cells, albeit their effects differed between HepG-2 and MDA-MB-231. CZIF-8 mostly induced G1phase arrest, whereas CZIF-8/HA caused a more pronounced G1 arrest in MDA-MB-231 cells and G1/S-phase accumulation in HepG-2. Even though, the in vitro results encourage us to study the effect of these particles in *vivo* either in treatment of tumor cells or to assay of their toxic effect on normal cells. These will help to elucidate the clinical suitability of this formulation of nanocomposites in the future.

## Data Availability

The data that support the findings of this study are available from the corresponding author upon reasonable request.
